# Phylogenetic Analysis of Bacterial Communities in Different Regions of the Gastrointestinal Tract of *Agkistrodon piscivorus*, the Cottonmouth Snake

**DOI:** 10.1371/journal.pone.0128793

**Published:** 2015-06-03

**Authors:** Timothy J. Colston, Brice P. Noonan, Colin R. Jackson

**Affiliations:** Biology Department, University of Mississippi, University, MS, United States of America; Wilfrid Laurier University, CANADA

## Abstract

Vertebrates are metagenomic organisms in that they are composed not only of their own genes but also those of their associated microbial cells. The majority of these associated microorganisms are found in the gastrointestinal tract (GIT) and presumably assist in processes such as energy and nutrient acquisition. Few studies have investigated the associated gut bacterial communities of non-mammalian vertebrates, and most rely on captive animals and/or fecal samples only. Here we investigate the gut bacterial community composition of a squamate reptile, the cottonmouth snake, *Agkistrodon piscivorus* through pyrosequencing of the bacterial 16S rRNA gene. We characterize the bacterial communities present in the small intestine, large intestine and cloaca. Many bacterial lineages present have been reported by other vertebrate gut community studies, but we also recovered unexpected bacteria that may be unique to squamate gut communities. Bacterial communities were not phylogenetically clustered according to GIT region, but there were statistically significant differences in community composition between regions. Additionally we demonstrate the utility of using cloacal swabs as a method for sampling snake gut bacterial communities.

## Introduction

Vertebrates are metagenomic organisms; they are not only composed of their own genetic material, but also that of their associated microbial communities [1]. The majority of these microorganisms are found in the host intestinal tract, and presumably assist in essential processes of energy and nutrient acquisition[2]. The ecological and evolutionary forces that act on both the host and it’s trillions of resident microorganisms sculpt the endogenic microbiome. With the advent of next generation sequencing technologies we are now better able than ever to characterize this observed microbial diversity. However, most studies investigating evolutionary patterns in vertebrate gut microbiomes have focused on mammals [1,2] and even among these studies, many have used captive animals from zoos or farms rather than wild populations. Very few studies have examined the gut microbiome of squamate reptiles (snakes, lizards), despite this being one of the most diverse and successful vertebrate clades.

The cottonmouth (*Agkistrodon piscivorus*, Serpentes, Viperidae) is a semiaquatic snake widespread throughout southeastern United States. The ecology and demographic history of *A*. *piscivorus* has been well studied, and it is often used as a model system in studies of venom evolution [3–6]. Though *A*. *piscivorus* is considered a generalist predator, preying upon reptiles, birds and invertebrates, the diet is dominated by amphibians and fish [6,7]. The paucity of information on the microbiome of wild vertebrate gastrointestinal tract (GIT) regions renders studies of any species meritorious, and exploration of the gut microbiome of *A*. *piscivorus* is particularly interesting as almost all other aspects of its ecology and biology are well known. Given the extent of knowledge on this organism’s natural history, inferences regarding factors influencing the composition of its GIT microbial community should be possible once that community has been well characterized. In this study we examine the bacterial communities of the small and large intestines, and cloaca of eight individuals of adult *A*. *piscivorus*. Our results reveal the presence of distinct bacterial community composition in each GIT region, provide novel insights into the diversity of squamate reptile associated bacterial communities in the wild, and demonstrate the utility of using non-lethal cloacal swabs to sample this diversity.

## Materials and Methods

### Ethics Statement

Adult snakes were collected and sacrificed in accordance with IACUC protocols approved by the Committee for Animal Care and Use at the University of Mississippi (#13–02 & #13–04). Euthanasia was performed using an overdose of the anesthetic lidocaine, injected into the brain (UM IACUC SOP #13–02). Collecting permits were obtained from the Mississippi Department of Wildlife, Fisheries, and Parks (permit #’s 0827101 & 1009112).

### Microbial Sampling

Snakes were sampled from two sites in Winston County (one individual, N32.98463 X W088.9980) and Lafayette County (seven individuals, N34.427238 X W089.38631), Mississippi, in spring of 2011 and spring of 2012 (Table 1). All snakes sampled were encountered during nighttime surveys along small streams leading to larger bodies of water. Snakes were collected by hand and safely restrained with clear plastic tubing placed over the head during sample collection. Once restrained, snakes were palpated to evaluate whether prey items were present in the GIT then the exterior cloaca of the snake was cleaned using a sterile alcohol pad. This sterilization step was to ensure that the cloacal sample primarily included cloaca associated microbes rather than environmental or transient microbes. Following cleaning, cloacal swabs were collected by inserting a sterile polyester-tipped applicator (Fisher Cat# 23-400-122) into the cloaca, taking care not to insert beyond the coprodeum and into the large intestine, then turning the swab several times before withdrawing. Once withdrawn the applicator was immediately placed into a sterile 2 ml tube and placed on ice before being transferred to a -20°C freezer prior to DNA extraction.

**Table 1:** Voucher numbers, locality information and region sampled for *Agkistrodon piscivorus* used in this study.

TJC Field ID #	OMNH Catalog #	County	Small Intestine	Large Intestine	Cloaca
103	44090	Winston	X	X	X
110	44088	Lafayette	X	X	X
111	44089	Lafayette	X	X	X
122	-	Lafayette	-	-	X
123	-	Lafayette	-	-	X
124	-	Lafayette	-	-	X
125	-	Lafayette	-	-	X
130	-	Lafayette	-	-	X

Three individuals (samples 103, 110, and 111) were sampled in more detail to determine the bacterial communities of their small and large intestines. These snakes were transported to the Department of Biology at the University of Mississippi where they were humanely euthanized. Immediately following death, a mid-ventral incision was made to expose the GIT, which was then removed. None of the individuals had identifiable prey items present in the GIT. Incisions were made in proximal and distal ends of both the small and large intestines, which were then swabbed with sterile polyester-tipped applicators that were immediately placed in sterile 2 ml collection tubes and frozen (-20°C) until DNA extraction. The remainder of the snake was preserved in 10% buffered formalin and whole specimens were deposited at the Sam Noble Oklahoma Museum of Natural History (Table 1).

### DNA Extraction

Microbial DNA was extracted by a bead beating procedure using MoBio Power Soil Extraction kits (MoBio Laboratories, Carlsbad, CA, USA). We followed the manufacturer’s standard DNA extraction protocol with minor adjustments. Thawed applicator tips were placed into bead tubes containing lysis buffer, and 50–100 μl of the lysis buffer was used to rinse any remaining particles that may have become dislodged from tips out of the 2ml collection tube and into the bead tube. Additional adjustments included incubating samples at 65°C for 15 min after the addition of Solution C1, and vortexing bead tubes horizontally for 25 min.

### PCR Amplification and Analysis

We used a nested PCR approach and bacterial specific primers to amplify a variable region of the 16S rRNA gene for initial analysis of the gut bacterial community by denaturing gradient gel electrophoresis (DGGE). This nested approach was necessary as DGGE can only be performed on fragments <500bp, and our template DNA was of low quantity rendering direct amplification of small fragments difficult. We first amplified near full-length fragments of the bacterial 16S rRNA gene using primer sets Bac8f and Univ1492r, and used this as our template for amplifying a shorter region for use in DGGE with the primer sets Bac1070f and Univ 1392GCr. Primers, PCR protocols, and cycle conditions have been previously described [8]. Amplification products from the second round of amplifications (bases 1070–1392) were analyzed in DGGE gels using a 40% to 70% denaturant gradient, and electrophoresis for 20 h at 80 V. Following electrophoresis, gels were stained with SYBR Green I and visualized by UV transillumination using a Kodak Gel Logic 200 system running Molecular Imaging Software 4.0 (Eastman Kodak, Rochester, NY, USA). Banding patterns were converted to binary data based on presence or absence of specific bands in each sample. Binary data was then used to create a distance matrix, showing similarity between samples (Yue & Clayton theta index [9]), and these relationships were visualized by ordination of samples through non-metric multidimensional scaling (NMDS). All analyses were performed using the bioinformatics software Mothur [10].

### Pyrosequencing

DGGE analyses revealed that the proximal and distal ends of the large and small intestines possessed near identical banding patterns, therefore we combined the DNA extraction from these regions such that each dissected individual had a single small and single large intestinal sample analyzed by pyrosequencing, along with the cloacal samples from both dissected and released individuals. The initial (bp 8–1492) 16S rRNA amplicons from each sample were sequenced using bacterial tag-encoded FLX amplicon 454 pyrosequencing (bTEFAP) [11] at the Research and Testing Laboratory sequencing facility (Lubbock, TX). Library amplification was performed with the bacterial primers 939f and 1392r [8,12] under the following conditions: 95°C for 5 minutes, followed by 35 cycles at 95°C for 30s, 54°C for 40s, an extension at 72°C for 1 minute, followed by a final elongation of 10 minutes at 72°C. Sequencing was performed on a Roche 454 FLX titanium instrument using standard reagents and following manufacturer’s guidelines. Sequences were deposited in the NIH NCBI Sequence Reads Archive (SRA Accession numbers SAMN03287547-SAMN03287560).

### Sequence Analysis

Pyrosequence data was accessed and processed in the program Mothur [10] following general procedures recommended by Schloss et al. [13]. Following denoising and barcode removal, sequences were aligned using the greengenes reference database (http://greengenes.secondgenome.com/, May 2013 version) and sequences differing by only a single nucleotide were grouped together. Sequences were checked for chimeras using the chimera.slayer command in Mothur and potential chimeras were removed. The resulting sequences were classified according to the greengenes database and any non-bacterial 16S sequences were removed. The remaining sequences were clustered into operational taxonomic units (OTUs) based on 97% similarity.

The distribution of OTUs in each sample was used for analyses of diversity and comparisons of community structure. Because samples varied in the number of final valid sequences obtained, all analyses were performed on a randomly sampled subset of the total dataset for each sample, which corresponded to the number of sequences in the smallest sample (i.e. all samples were standardized to be equivalent to the sample with the lowest number of valid reads). This random subsampling was performed 1,000 times for each analysis, with the composite outcome reported. Alpha diversity within each sample was determined by Schao and inverse Simpsons indices, and rarefaction and collection curves were used to visualize whether our procedures included enough sampling (enough reads) to assess this diversity. Beta diversity (comparisons of bacterial community structure between samples) was examined using the Yue & Clayton theta similarity index [9] which accounts for proportional abundance of OTUs in a sample. Similarity between samples was visualized by NMDS, Venn diagrams, as well as dendrogram construction. We tested the spatial separation of samples observed in NMDS through analysis of molecular variance (AMOVA) and analysis of similarities (ANOSIM). Dendrograms were constructed based on Yue & Clayton theta (thetayc) distances and a 95% majority rule consensus tree was generated from the distribution of 1000 trees without burn in using the program TreeAnnotator [14].

## Results

### DGGE Analyses

**Fig 1:**
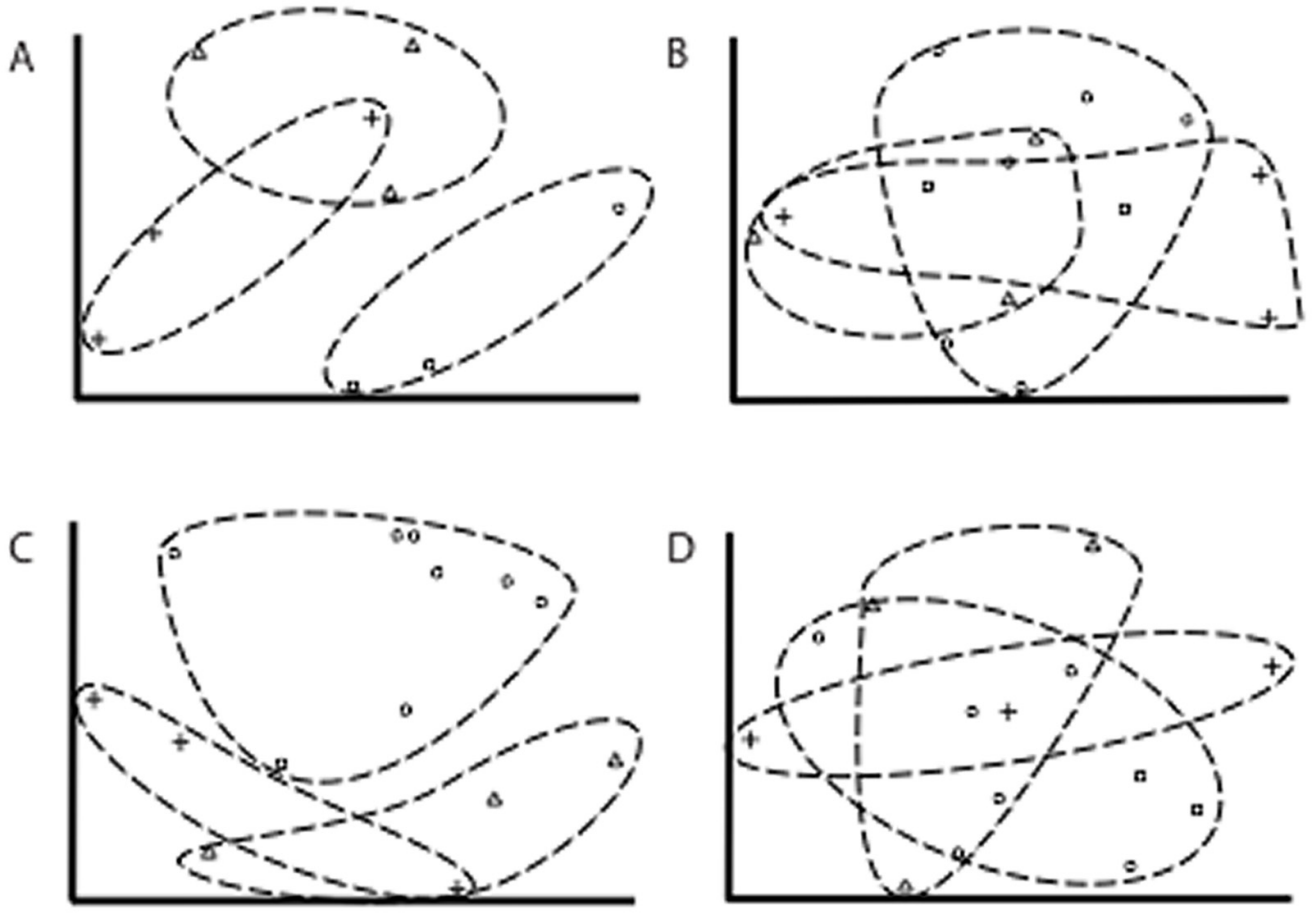
Nonmetric multidimensional plots of bacterial communities. Nonmetric multidimensional plots (three dimensions, stress <0.2; the first two dimensions are shown) based on Yue & Clayton theta (thetayc) similarities of bacterial communities from the small intestine (triangles), large intestine (plus signs), and cloaca (circles) of *Agkistrodon piscivorus*. Plots are determined from: A) DDGE profiles, B) all OTUs recovered from 454 sequencing, C) dominant OTUs recovered from 454 sequencing (rarest 1% of sequences removed), D) very dominant OTUs recovered from 454 sequencing (rarest 10% of sequences removed). Dashed ellipses indicate groupings based on region of the GIT.

While based on a limited number of samples from just two locations, NMDS of community similarity based on DGGE binary data showed a clear pattern of distinct small intestine, large intestine, and cloacal communities, with only slight overlapping of multidimensional space between the large and small intestine samples (Fig 1A). There was no apparent association in GIT bacterial community structure among individuals or localities, although our ability to test this was limited by the number of individuals sampled. All regions of the GIT show similar levels of diversity and richness as inferred from DGGE banding patterns. Band number, used as a proxy for richness [15], ranged from 8–29, with a mean of 17.

### Sequence Analyses

After alignment, gap removal, and potential chimera removal we recovered 40,317 valid sequence reads, representing 7,137 unique sequences with a median length of 267 bp from our pooled dataset. The classified sequences were binned into OTUs and rarefaction suggested that we recovered >98% of the diversity in all but one of our samples, and with that sample (individual 103l) we recovered 95% of the diversity (Table 2).

**Table 2:** Statistical analyses of bacterial 16S rRNA gene pyrosequence data obtained from the gastrointestinal tract (GIT) of individual (ID) *Agkistrodon piscivorus* (numbered 103–130).

ID	103 (c)	103 (l)	103 (s)	110 (c)	110 (l)	110 (s)	111 (c)	111 (l)	111 (s)	122 (c)	123 (c)	124 (c)	125 (c)	130 (c)
***Coverage***	99.22%	95.45%	99.03%	98.91%	99.49%	99.64%	99.14%	99.26%	99.70%	98.31%	99.02%	98.92%	99.26%	99.17%
**S**_**obs**_	40	143	52	28	31	15	68	51	110	64	63	55	54	29
**S**_**Chao1**_	63.8	208	80.9	58.3	109	29	105.5	59.23	161.7	88.4	84.4	76.9	67.2	32.5
**Inverse Simpsons index**	4.575	11.559	4.471	2.497	3.069	1.085	10.489	7.231	13.940	8.599	8.983	7.918	5.961	4.448

Different regions of the GIT are designated as (c) cloaca (l) large intestine (s) small intestine. Inverse Simpson’s diversity index and Schao were used to assess alpha diversity and compare species diversity between samples. **S**_**obs**_ **=** observed number of species.

**Fig 2:**
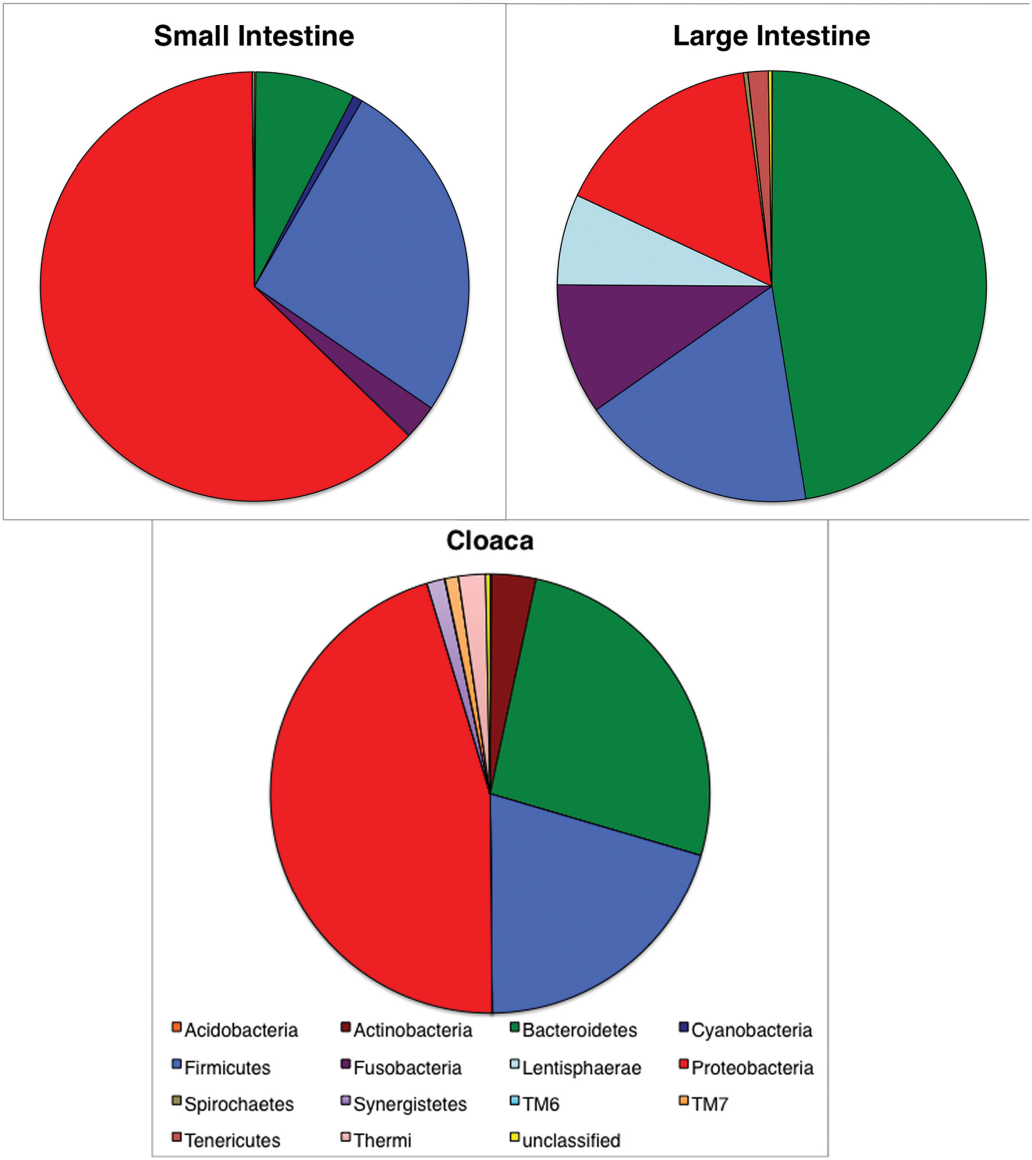
*Agkistrodon piscivorus* GIT bacterial abundance. Relative abundance of major bacterial lineages found in various regions of the gastrointestinal tract of all *Agkistrodon piscivorus* as identified 16S rRNA gene pyrosequencing. Proportions represent the proportion of 454 sequence reads classified as being in that taxon (number of reads ranges from 5,620–14,905).

Bacterial diversity recovered in our 7,137 unique sequences was binned into 503 distinct OTUs spanning 14 bacterial phyla, with <0.002% (just 92 sequences) of the 40,317 sequence dataset designated as “unclassified Bacteria”. Among individuals for which all three regions were sampled the large intestine harbored the most diverse bacterial communities in two of the three individuals (Table 2). In terms of community composition, the large intestine samples were dominated by sequences affiliated with the Bacteroidetes, followed by Firmicutes, Proteobacteria, and Lentisphaerae, while members of the Proteobacteria were the dominant group in both the small intestine and cloaca samples, followed by sequences classified as Firmicutes and Bacteroidetes (Fig 2). Gammaproteobacteria were the dominant subphylum of Proteobacteria in all but two samples; one small intestine (110) sample and one cloacal sample (111) had Deltaproteobacteria and Betaproteobacteria, respectively, as their dominant Proteobacteria. Importantly, the dominant bacterial phyla found in both the small and large intestines were also found in cloacal samples (Fig 2).

NMDS plots based on pyrosequence data did not show as tight of a grouping of samples by GIT region as was produced by DGGE analyses (Fig 1A and 1B). ANOSIM and AMOVA tests of spatial separation did suggest a significant difference in community composition among all three regions as a group (AMOVA: df = 2 F_s_ = 1.902 p = 0.005, ANOSIM: R^2^ = 0.606 p = 0.001), but none of the individual pairwise comparisons between different GIT regions were significant. Next generation sequencing is likely to detect ultra-rare species that are typically not detected by DGGE [16], and these may not have an important role in the GIT community, or could represent transitory cells that are not permanent members of the gut community. Therefore we also analyzed the pyrosequence data after sequentially removing the rarest 1%, 5% and 10% of sequences. NMDS of communities with the rarest 1% and 5% of sequences removed resembled the structuring seen in our DGGE data, although that signal was lost once the rarest 10% of sequences are removed (Fig 1C (1%), D (10%), 5% removed not shown in figure for clarity). AMOVA following the removal of the rarest 5% of sequences still suggested overall community differences among GIT regions (p = 0.007), without any individual pairwise comparisons between GIT regions being significant. However, ANOSIM did detect significant differences for all pairwise comparisons of GIT regions once any level (1%, 5%, or 10%) of the rarest sequences was removed (Table 3).

**Table 3:** Results of ANOSIM analyses comparing the bacterial communities in different regions of the GIT of individuals of *Agkistrodon piscivorus*. GIT regions are the large intestine (L), small intestine (S), cloaca (C).

Region	1% Removed		5% Removed		10% Removed	
	R^2^	p	R^2^	p	R^2^	p
C-L-S	0.614	0.001	0.469	0.002	0.441	0.005
C-L	0.683	0.006	0.524	0.012	0.511	0.012
C-S	0.718	0.006	0.551	0.011	0.546	0.011

Analyses were performed with different levels of the rare sequences removed (<1%, <5%, or <10% of total reads).

**Fig 3:**
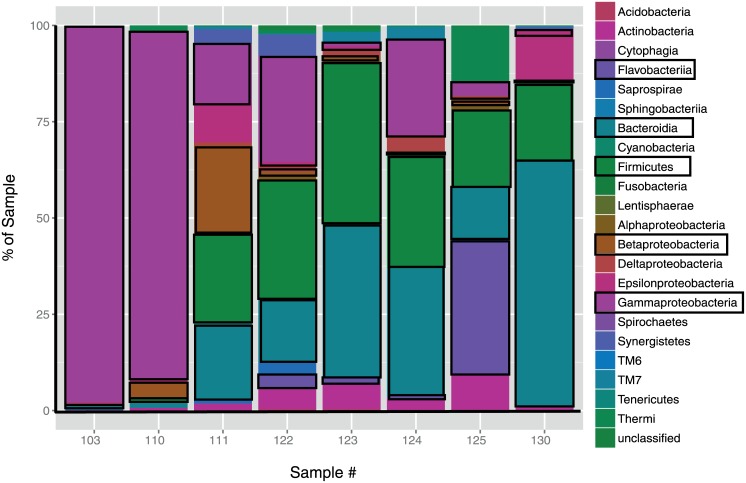
Bacterial abundance present in the cloaca of *Agkistrodon piscivorus*. Relative abundance of bacterial lineages found in cloacal samples of *Agkistrodon piscivorus* individuals (numbered) as identified 16S rRNA gene pyrosequencing. Abundances are based on proportional number of 454 reads (from 839–2919) total reads per sample). Major bacterial groups are outlined by black boxes.

Because we had cloacal samples from both dissected and released specimens, we focused on the cloaca as our primary GIT region of interest, as this would allow future sampling to avoid sacrificing individuals. Furthermore, of the 22 OTUs representing greater than 1% of the sequence reads, all but four were found in the cloaca as well as other regions (Table 4). Based on proportions of sequence reads obtained, the most abundant bacterial lineage in the cloaca varied among individuals: Members of the Bacteroidetes were dominant in two samples, Proteobacteria (primarily subphyla Gamma and Beta) were dominant in two, similar levels of abundance of Bacteroidetes and Firmicutes were found in three, while one sample had similar proportions of Bacteroidetes, Proteobacteria and Firmicutes (Fig 3). At a finer taxonomic scale, the most abundant sequences affiliated with Bacteroidetes were classified as members of the genus *Bacteroides*, with two different species dominating in the large intestine and cloaca (Table 4). While the most abundant Proteobacteria (specifically Gammaproteobacteria) sequences obtained from the cloaca were classified as either *Salmonella enterica*, or as unclassified Enterobacteriaceae, those of the small intestine were identified as *Pseudomonas veronii* and Aeromanadaceae (Table 4). Both *Enterobacter* and *Acinetobacter* were found in all regions of the GIT. The most numerous Firmicutes sequences were in the family Clostridiaceae and those we were able to classify to genus were *Clostridium* (Table 4).

**Table 4:** Distribution and classification of 16S rRNA-defined bacterial OTUs representing >1% of the total proportion of reads obtained from the GIT of individuals of *Agkistrodon piscivorus*.

Phylum/Subphylum	Finest Taxonomic Classification	% of total	Region
Bacteroidetes	*Bacteroides sp*.	10.07	LS
Gammaproteobacteria	Enterobacteriaceae	7.50	LSC
Deltaproteobacteria	Desulfovibrionaceae	6.40	LS
Gammaproteobacteria	Enterobacteriaceae	5.34	LSC
Bacteroidetes	Porphyromonadaceae	5.28	LSC
Gammaproteobacteria	*Salmonella enterica*	4.61	LSC
Gammaproteobacteria	*Pseudomonas veronii*	4.23	LSC
Gammaproteobacteria	Aeromonadaceae	3.25	LSC
Fusobacteria	*Cetobacterium somerae*	3.18	LSC
Firmicutes	Clostridiaceae	2.99	LSC
Firmicutes	Peptostreptococcaceae	2.98	LSC
Gammaproteobacteria	*Enterobacter sp*.	2.37	LSC
Gammaproteobacteria	*Acinetobacter sp*.	2.00	LSC
Firmicutes	*Clostridium sp*.	1.87	LSC
Firmicutes	Clostridia	1.79	C
Betaproteobacteria	*Janthinobacterium lividum*	1.63	S
Gammaproteobacteria	*Edwardsiella sp*.	1.56	S
Bacteroidetes	Weeksellaceae	1.54	C
Bacteroidetes	*Bacteroides sp*.	1.21	C
Epsilonproteobacteria	*Campylobacter fetus*	1.14	C
Gammaproteobacteria	Enterobacteriaceae	1.07	LSC
Betaproteobacteria	*Achromobacter sp*.	1.06	C

Region designates the region of the GIT where that OTU was detected: large intestine (L), small intestine (S), cloaca (C).

**Fig 4:**
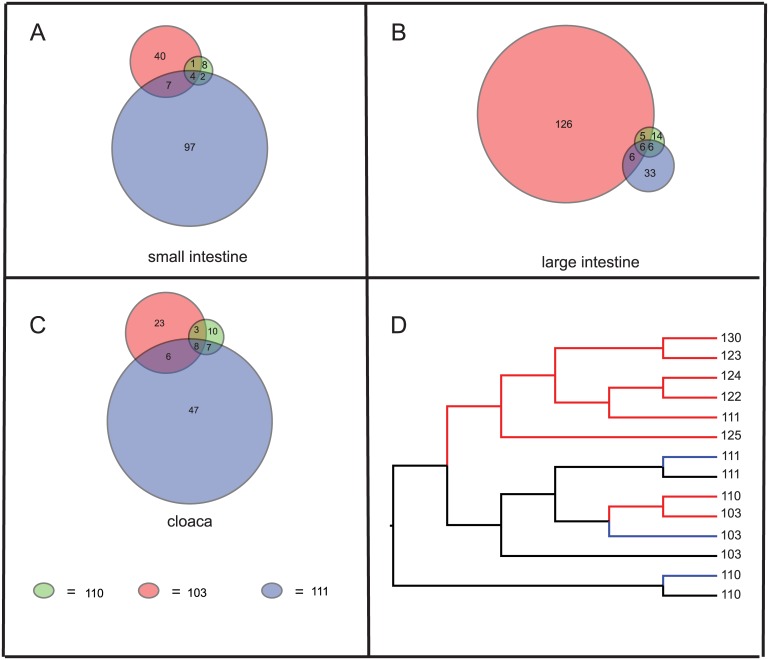
Bacterial community similarity by region and phylogenetic reconstruction of bacterial communities found in Agkistrodon piscivorus. Graphical representation of bacterial community similarity in GI regions sampled from *Agkistrodon piscivorus* individuals: A- C) Venn diagrams of small intestine (A), large intestine (B) and cloacal (C) samples from the three individuals sampled destructively (103, 110, 111). Circles are drawn such that the area of the circle is proportional to the number of OTUs found in each region. Numbers represent the number of OTUs either shared or specific to that individual. D) 95% majority rule consensus tree based on Yue & Clayton theta distances for bacterial communities in all individuals sampled (numbered) with branches colored by sample region (blue = small intestine, black = large intestine, red = cloacal).

Venn diagrams revealed that some OTUs in each region of the GIT were shared by all three individuals sampled, but the majority of OTUs in each region were unique to a given individual (Fig 4). Dendrogram analysis did not reveal a definitive pattern of clustering by GIT region sampled or individual, although cloacal samples tended to group together (Fig 4).

## Discussion

The aim of this study was to characterize the gut microbiome of a non-captive squamate reptile species. Only one other study has examined bacterial community structure in the GIT of wild snakes [17]. That study included two individuals of *A*. *piscivorus*, the species studied here, but the authors focused on the most abundant bacterial populations in the gut community as determined from DGGE followed by traditional Sanger sequencing [17], rather than incorporating the high throughput sequencing approach that we used. Despite the difference in methodology, that study also found the Firmicutes and Bacteroidetes to be the most dominant phyla in the GIT of *A*. *piscivorus*, although no samples of the cloaca were taken and, presumably because of methodological constraints, the study did not attempt to assess diversity or abundance.[17]. Our study demonstrates the utility of both standardized sampling via cloacal swabs and the greater information in community composition that can be obtained by using next generation sequencing rather than traditional methods of bacterial community analyses.

Both DGGE and next generation sequencing recovered distinct bacterial communities corresponding to discrete regions of the GIT (small intestine, large intestine, cloaca), although this was only apparent when the more common sequence types were considered. As might be expected given the cloaca’s terminal location, cloacal samples captured the bacterial diversity found in earlier regions of the GIT, but not necessarily the proportional abundance of specific taxa. While the dominance of members of the Bacteroidetes in the large intestine is consistent with other findings in snakes [17,18], the dominance of members of the Proteobacteria in the small intestine and cloaca was unexpected. Previous work has suggested that the small intestine of captive snakes is dominated by members of the Firmicutes and Bacteroidetes, regardless of physiological processes (e.g. active digestion) [18]. Our findings suggest this may not be the case in wild snakes. It is well known that the mammalian gut is dominated by members of the Bacteroidetes and Firmicutes [19] and the limited amount of previous work on wild reptiles (e.g. marine iguanas) and captive snakes suggests that this is the same for reptiles [18,20]. However, a recent meta-analysis compared the GIT bacteria of insects, birds and mammals and found that the bacterial community in insects and birds is more likely to be dominated by Proteobacteria and Firmicutes rather than Bacteroidetes [21]. The dominance of Proteobacteria in the GIT of snakes in this study suggests that predatory snakes may show more similarities to birds in terms of their GIT bacterial communities than to other vertebrate organisms.

In one of the few previous studies to investigate bacterial community structure in wild squamates, Hong et al. found that sequences affiliated with the Firmicutes dominated fecal samples from both marine and land iguanas in the Galapagos, with the classes Bacteroidia and Clostridia being dominant in both host species [20]. They suggested that the similarity between these herbivorous reptiles to mammals in the general composition of their GIT bacterial assemblages is because of the prevalence of herbivory in mammals, and that abundance of members of the Bacteroidetes and Firmicutes in the gut aids in the breakdown and nutrient acquisition of complex polysaccharides [2,20]. Wood eating fish species have also been shown to contain similar GIT diversity to herbivorous mammals [22] and these similarities (at the level of phylum) have led some authors to suggest that fish served as the original vertebrate hosts to these bacterial communities [23,24]. Although *A*. *piscivorus* is a predatory species (no snakes are herbivorous), Clostridia were the dominant Firmicutes recovered in our analysis, possibly reflecting a broader function of this subset of Firmicutes in the GIT that is not restricted to herbivory. It may be that this class of obligate fermentative bacteria is so well adapted to the anoxic conditions of the intestines that they are likely to be dominant in the GIT of any larger organism. Cottonmouth snakes are generalist predators and are known to feed on carrion; and some authors suggest that certain populations of *A*. *piscivorous* my acquire food primarily by scavenging [25]. Many Clostridia are associated with the fermentation of animal material and this may convey benefits to host species that prey on or scavenge other vertebrates.

All of the individuals sampled in our study were adults, and no detectable prey items were found in dissected individuals or through palpitation of non-dissected individuals. That said, the broad variation in dominant bacterial OTUs we recovered from cloacal samples leads us to suspect that abundance may be influenced by active digestion of prey items that were undetected. Although the bacterial species present in the GIT has been shown to remain constant throughout digestion and subsistence in captive snakes, the relative abundance of these species can be correlated with active physiological processes such as digestion [18]. An additional factor relating to diet variation in the gut bacterial community that has yet to be explored is ontogenetic change. Diet has been shown to change through ontogeny in *A*. *piscivorus* [26], and it’s not unreasonable to suspect that this could lead to changes in bacterial community structure. Acquisition of a new diet has been hypothesized to be a fundamental driver for species’ diversification and concomitant gut microbiota evolution [19] and dietary changes during ontogeny should also drive changes in gut microbiota. Lastly, it has been shown that in mammalian hosts which undergo torpor during hibernation both bacterial community composition and richness vary dramatically during hibernation [27]. It would be interesting to explore whether this holds true for ectothermic species that undergo hibernation, which *A*. *piscivorus* does in certain areas of its range.

Our study lays the groundwork for further investigation of gut bacterial community structure in squamate reptiles. We identified discrete bacterial communities that correspond to regions along the GIT and show that cloacal samples encompass the breadth of bacterial diversity found in the snake gut. Additionally we show the utility of our standardized sampling method using cloacal swabs. Interestingly, and in contrast to previous investigations of snakes, we find that at the phylum level the snake gut microbiome shows more similarities to that of birds than to other vertebrates. Future studies should include broader sampling of host species for more detailed comparative analyses, and test whether gut bacterial communities function as either evolutionary or ecological traits.

## References

[1] Ley RE, Lozupone CA, Hamady M, Knight R, Jeffrey I (2009) Worlds within worlds: evolution of the vertebrate gut microbiota. Nat Rev Microbiol 6: 776–788. 10.1038/nrmicro1978 PMC2664199 18794915

[2] Ley RE, Hamady M, Lozupone C, Turnbaugh P, Roy R, Bircher JS, et al (2009) Evolution of mammals and their gut microbes. Science (80-) 320: 1647–1651. 10.1126/science.1155725 PMC2649005 18497261

[3] Guiher TJ, Burbrink FT (2008) Demographic and phylogeographic histories of two venomous North American snakes of the genus Agkistrodon. Mol Phylogenet Evol 48: 543–553. 10.1016/j.ympev.2008.04.008 18539486

[4] Roth ED (2005) Spatial Ecology of a Cottonmouth (Agkistrodon piscivorus) Population in East Texas. J Herpetol 39: 312–315.

[5] Lomonte B, Tsai W-C, Ureña-Diaz JM, Sanz L, Mora-Obando D, Sánchez EE, et al (2014) Venomics of New World pit vipers: Genus-wide comparisons of venom proteomes across Agkistrodon. J Proteomics 96: 103–116. 10.1016/j.jprot.2013.10.036 24211403 PMC4294458

[6] Vincent SE, Herrel A, Irschick DJ (2004) Sexual dimorphism in head shape and diet in the cottonmouth snake (Agkistrodon piscivorus). J Zool 264: 53–59.

[7] Ford NB (1997) Ecology of the Western Cottonmouth (Agkistrodon piscivorus leucostoma) in Northeastern Texas. Biology of the Vipers. Vol. 1987 pp. 167–178.

[8] Jackson CR, Langner HW, Donahoe-Christiansen J, Inskeep WP, McDermott TR (2001) Molecular analysis of microbial community structure in an arsenite-oxidizing acidic thermal spring. Environ Microbiol 3: 532–542. 11578314 10.1046/j.1462-2920.2001.00221.x

[9] Yue JC, Clayton MK (2005) A Similarity Measure Based on Species Proportions. Commun Stat Theory Methods 34: 2123–2131.

[10] Schloss PD, Westcott SL, Ryabin T, Hall JR, Hartmann M, Hollister EB, et al (2009) Introducing mothur: open-source, platform-independent, community-supported software for describing and comparing microbial communities. Appl Environ Microbiol 75: 7537–7541. 10.1128/AEM.01541-09 19801464 PMC2786419

[11] Dowd SE, Callaway TR, Wolcott RD, Sun Y, McKeehan T, Hagevoort RG, et al (2008) Evaluation of the bacterial diversity in the feces of cattle using 16S rDNA bacterial tag-encoded FLX amplicon pyrosequencing (bTEFAP). BMC Microbiol 8: 125 10.1186/1471-2180-8-125 18652685 PMC2515157

[12] Baker GC, Smith JJ, Cowan DA (2003) Review and re-analysis of domain-specific 16S primers. J Microbiol Methods 55: 541–555. 14607398 10.1016/j.mimet.2003.08.009

[13] Schloss PD, Gevers D, Westcott SL (2011) Reducing the effects of PCR amplification and sequencing artifacts on 16S rRNA-based studies. PLoS One 6: e27310 10.1371/journal.pone.0027310 22194782 PMC3237409

[14] Rambaut A, Drummond AJ (2007) TreeAnnotator (v 1.5). Available: http://beast.bio.ed.ac.uk./TreeAnnotator.

[15] Jackson CR, Churchill PF, Roden EE (2001) Successional changes in bacterial assemblage structure during epilithic biofilm development. Ecology 82: 555–566.

[16] Caporaso JG, Lauber CL, Walters WA, Berg-Lyons D, Lozupone CA, Turnbaugh PJ, et al (2011) Global patterns of 16S rRNA diversity at a depth of millions of sequences per sample. Proc Natl Acad Sci U S A 108 Suppl: 4516–4522. 10.1073/pnas.1000080107 20534432 PMC3063599

[17] Hill JG III, Hanning I, Beaupre SJ, Ricke SC, Slavik MM (2008) Denaturing gradient gel electrophoresis for the determination of bacterial species diversity in the gastrointestinal tracts of two crotaline snakes. Herpetol Rev 39: 433–438.

[18] Costello EK, Gordon JI, Secor SM, Knight R (2010) Postprandial remodeling of the gut microbiota in Burmese pythons. ISME J 4: 1375–1385. 10.1038/ismej.2010.71 20520652 PMC3923499

[19] Ley RE, Lozupone CA, Hamady M, Knight R, Gordon JI (2008) Worlds within worlds: evolution of the vertebrate gut microbiota. Nat Rev Microbiol 6: 776–788. 10.1038/nrmicro1978 18794915 PMC2664199

[20] Hong P-Y, Wheeler E, Cann IKO, Mackie RI (2011) Phylogenetic analysis of the fecal microbial community in herbivorous land and marine iguanas of the Galápagos Islands using 16S rRNA-based pyrosequencing. ISME J 5: 1461–1470. 10.1038/ismej.2011.33 21451584 PMC3160690

[21] Hird SM, Carstens BC, Cardiff SW, Dittmann DL, Brumfield RT (2014) Sampling locality is more detectable than taxonomy or ecology in the gut microbiota of the brood-parasitic Brown-headed Cowbird (Molothrus ater). PeerJ 2: e321 10.7717/peerj.321 24711971 PMC3970801

[22] McDonald R, Schreier HJ, Watts JEM (2012) Phylogenetic Analysis of Microbial Communities in Different Regions of the Gastrointestinal Tract in Panaque nigrolineatus, a Wood-Eating Fish. PLoS One 7: e48018 10.1371/journal.pone.0048018 23133540 PMC3485024

[23] Sullam KE, Essinger SD, Lozupone CA, O’Connor MP, Rosen GL, Knight R, et al (2012) Environmental and ecological factors that shape the gut bacterial communities of fish: a meta-analysis. Mol Ecol 21: 3363–3378. 10.1111/j.1365-294X.2012.05552.x 22486918 PMC3882143

[24] Clements KD, Angert ER, Montgomery WL, Choat JH (2014) Intestinal microbiota in fishes: what’s known and what's not. Mol Ecol 23: 1891–1898. 10.1111/mec.12699 24612310

[25] Devault TL, Krochmal AR (2002) Scavenging by snakes: an examination of the literature. Herpetologica 58: 429–436.

[26] Vincent SE, Herrel A, Irschick DJ (2004) Ontogeny of intersexual head shape and prey selection in the pitviper Agkistrodon piscivorus. Biol J Linn Soc 81: 151–159.

[27] Dill-McFarland KA, Neil KL, Zeng A, Sprenger RJ, Kurtz CC, Suen G, et al (2014) Hibernation alters the diversity and composition of mucosa-associated bacteria while enhancing antimicrobial defence in the gut of 13-lined ground squirrels. Mol Ecol 23: 4658–4669. 10.1111/mec.12884 25130694

